# Effect of ciliary-muscle contraction force on trapezius muscle activity during computer mouse work

**DOI:** 10.1007/s00421-018-4031-8

**Published:** 2018-11-14

**Authors:** Dmitry Domkin, Mikael Forsman, Hans O. Richter

**Affiliations:** 1 Centre for Musculoskeletal Research, Department of Occupational and Public Health Sciences, Faculty of Health and Occupational Studies, University of Gävle, 80176 Gävle, Sweden; 2 Institute of Environmental Medicine, Karolinska Institutet, Stockholm, Sweden; 3 KTH Royal Institute of Technology, Stockholm, Sweden

**Keywords:** Eye-accommodation, Ciliary-muscle contraction force, Computer mouse work, Electromyography, Trapezius muscle, Visual ergonomics

## Abstract

The present study aimed to identify whether or not an increase in ciliary-muscle contraction force, when the eye-lens is adjusted for viewing at a near distance, results in an increase in trapezius muscle activity, while performing a natural work task. Twelve participants, ranging in age from 21 to 32 years, performed a computer-mouse work task during free gaze conditions. A moving visual target was tracked with a computer mouse on a screen placed at two different distances from the eyes, 25 cm and 50 cm. Tracking performance, eye accommodation, and bilateral trapezius muscle activity were measured continuously. Ciliary-muscle contraction force was computed according to a formula which takes into account the age-dependent, non-linear relationship between the contraction force of the ciliary muscle and the produced level of eye accommodation. Generalized estimating equations analyses were performed. On the dominant hand side and for the nearest screen distance, there was a significant effect of ciliary-muscle contraction force on the trapezius muscle activity (*p* < 0.001). No other effects were significant (*p* > 0.05). The results support the hypothesis that high visual demands, during computer mouse work, increase ciliary muscle contraction force and contribute to a raise of the sustained level of trapezius muscle activity. The current study specifically clarifies the validity of the relationship between ciliary-muscle contraction force and trapezius muscle activity and demonstrates that this relationship is not due to a general personality trait. We conclude that a high level of ciliary muscle contraction force can contribute to a development of musculoskeletal complaints in the neck–shoulder area.

## Introduction

A prerequisite for a stable gaze and the ability to foveate a target (e.g., an alphanumerical character) is sensorimotor synergy between the extraocular muscles that move eyes and those muscles that stabilize the position of the head. Previous research has demonstrated that not only eye movements, but also eye-lens accommodation is integrated by neck muscle function (Richter et al. 2010). This integration coordinates movements of the eyes and head, and thereby stabilizes gaze (Richter et al. 2011). Eye-lens accommodation is achieved by contraction of the ciliary muscle, an intraocular muscle that regulates the focal point of the eye-lens, adjusting it to objects at different distances. Previous studies (Richter 2014; Richter et al. 2010, 2011; Zetterberg et al. 2013) that applied indirect optometric measures of eye-lens accommodation, have demonstrated a relationship between accommodation/vergence load and neck muscle activation level (trapezius muscle). According to the “Cinderella hypothesis” (Hägg 1991), a sustained low-load muscle activity can be a risk factor for musculoskeletal complaints. Strenuous visual near work with a high level of eye-lens accommodation may, according to the Cinderella hypothesis, result in long-lasting tension within the neck muscles. The consequence is the development of chronic neck pain due to unfavorable visual and postural ergonomic conditions. Currently, the oculomotor connection between high visual demand and neck muscle activity is not well understood.

The mentioned studies by Richter (2014), Richter et al. (2010, 2011) and Zetterberg et al. (2013) were the first to objectively and continuously measure eye-lens accommodation while simultaneously measuring trapezius muscle activity. However, those studies were conducted in laboratory settings that were different from typical working conditions. They were conducted under stationary conditions, with the eyes continuously focused on a completely stationary object and without a concurrent natural motor task. Different levels of eye-lens accommodation and induced eye strain were achieved artificially by adding external optical trial lenses of varying strengths. The limitations of those studies have been addressed in a recent study by Domkin et al. (2016). In that study, the relationship between ciliary-muscle contraction force and trapezius muscle activity was studied under free gaze conditions and during a dynamic natural working task. The main finding was a strong and significant correlation between the level of ciliary-muscle contraction force and the level of trapezius muscle activity in individuals ranging in age from 19 to 28 years. That is, participants who had higher levels of ciliary-muscle contraction force generally also had higher levels of trapezius muscle activity. What remained unanswered was whether the correlation between ciliary-muscle contraction force and trapezius muscle activity was a genuine causal effect of visual load on trapezius muscle activity or whether the correlation was associative in nature and due to individual traits.

In our previous study (Domkin et al. 2016), the participants performed a tracking motor task similar to drawing on a touch screen and holding a pen without arm support on the active side. In the present study, a visuomotor task was performed that resembled office computer work, with participants looking at a vertical screen and operating a computer mouse placed on a table.

The aim of the present study was to answer the following research questions:

1. Does the increase in ciliary-muscle contraction force, when the eye-lens is adjusted for viewing at a near distance, result in an increase in trapezius muscle activity, while performing a natural work task? The hypothesis was that the magnitude of eye-lens accommodation to a visual target at a near distance correlates with a concurrent increase in trapezius-muscle activity level.
2. Do personal traits influence the relationship between ciliary-muscle contraction force and trapezius muscle activity (Domkin et al. 2016)? We hypothesized that if such traits exist then they will be manifest at differing levels of eye-lens accommodation for near distance viewing.

To achieve conditions in which the effect of ciliary-muscle contraction force on trapezius muscle activity will manifest within an individual, two different viewing distances for near viewing were employed. These produced two distinct levels of eye-lens accommodation and consequently, two different levels of ciliary-muscle contraction force within an individual.

## Methods

### Participants

Twelve right-handed individuals, the majority of them university students, participated in this study with the following characteristics: age 26 ± 3 years (mean ± SD), five men and seven women, with normal, or corrected to normal, vision, and without musculoskeletal complaints in the neck, shoulders, arms, or hands. Individuals < 40 years of age were enrolled to ensure a large range of accommodation. The study conformed to the standards set by the Declaration of Helsinki. The Regional Ethics Review Board approved the study (2014:366). All participants gave their written informed consent before participation.

### Testing procedure

The participants sat at a table placed in a darkened room, with their head resting on a support for the chin and forehead that restricted head movement. The height of their head position was adjusted to keep the sight line on the same level for all participants (a requirement of the PlusOptix power refractor, see below), the height of the monitor was constant. A computer screen (Dell U2311Hb 23-inch monitor with a resolution of 1920 × 1080 pixels) was placed vertically in landscape orientation on the table in front of the participants. The participant’s task was to track a low contrast, slowly moving visual target within the central screen area (5 × 5 cm) with the computer mouse cursor, which participants operated with their right hand on the table surface. The target could move in any direction within the central area. The mouse was a standard wired optical 3-button computer mouse for office usage. The mouse cursor was of a standard arrow shape. The screen coordinates of the mouse cursor were continuously sampled at 60 Hz with a resolution of 0.25 mm. There were two tracking task conditions, one with the screen placed at a “near” distance of 25 cm (4 diopters) from the participant and another with the screen placed at a “far” distance of 50 cm (2 diopters). The order of condition presentation was random. The duration of each tracking task condition was 10 min. There was a 5-min pause between conditions. The tracking target was a circle of ca 0.7° visual angle (ca 3 mm in diameter for the “near” and ca 6 mm for the “far” condition), moving at a speed of 5 mm/s along a pseudo-random, pre-computed unpredictable trajectory. The participants were instructed to track the target keeping the mouse cursor tip in the middle of the target circle.

For optimal accommodation stimulation, the spatial frequency of the circle’s gray shading square-wave grating was set to 7 cycles/deg (Owens 1980). The square-wave gratings of the tracking target were inclined at 45° clockwise from the vertical. Along with the tracking target, three other non-target circles with different inclinations of grating (vertical, horizontal, and 45° counter clockwise relative to the vertical) were moving on the screen in the vicinity of the target circle along pseudo-random, pre-computed, unpredictable trajectories. In the constant presence of the three non-target circles with different inclinations of grating, the participants had to continuously maintain a task-relevant level of eye-lens accommodation on the screen to distinguish and track the target circle [see Domkin et al. (2016) for an image of the circles]. In addition, a standardized task instruction emphasized active accommodation: “Focus on the moving target and carefully track it with the cursor at all times”. The movement speed of non-target circles varied around 4.8 ± 3.2 mm/s (mean ± SD). The difference in average speed of movement of the tracking target and non-targets were due to the method of computation of the movement trajectory as concatenated arcs [cf. Domkin et al. (2013)]. This small difference in speed probably acted to make the tracking task even more difficult because it forced participants to pay close attention and focus accommodation harder on the target.

From continuously sampled coordinates of the cursor and of the tracking target, a measure of tracking performance (tracking error) was computed as the distance between the cursor tip and the center of the target circle. Tracking error served as an indicator of task compliance.

### Accommodative response and ciliary-muscle contraction force

Participant’s eye refraction was measured continuously during the tracking task using an infrared photorefraction camera with a sampling frequency of 25 Hz and a precision of ± 0.25 diopter (PlusOptix power refractor II S04, PlusOptix GmbH, Germany). The photorefractor had been calibrated and validated [see Richter et al. (2010)]. In general, measurements with PlusOptix system photorefractors have been validated in the scientific literature (Allen et al. 2003; Hunt et al. 2003). The PlusOptix photorefraction camera records refraction values in relation to a 1-m reference distance, which has a dioptric value of zero. The recorded refraction values were converted to diopter values of accommodative response with zero value if focusing at infinite (∞) distance: *D* = 1 – *R*, where *D* is accommodative response and *R* is recorded refraction [cf. Richter et al. (2010)]. The mean accommodative response for each minute of the task was computed for each eye.

Ciliary-muscle contraction force was computed from eye-lens accommodative responses according to the formula suggested by Fisher (1977). This computation takes into account the age-dependent, non-linear relationship between contraction force of the ciliary muscle and the produced level of eye accommodation:

$$F={\left( {\frac{D}{K}} \right)^2}$$

where *F* is the ciliary-muscle contraction force (mN), *D* is the accommodative response (diopters) and *K* is the age-dependent dioptric force coefficient that is computed as $K=0.675 - 0.02 \times {\text{age}}+0.000147 \times {\text{ag}}{{\text{e}}^2}.$

The averaged across minutes ciliary-muscle contraction force (CMCF) values for the right eye and left eye were not normally distributed (Shapiro–Wilk test of normality *p* < 0.05). The Spearman rank correlation coefficient was, therefore, computed between the right and left CMCF values: *r* = 0.71, *p* < 0.01 for “near” distance, and *r* = 0.8, *p* < 0.01 for “far” distance. There was no significant difference in CMCF values between right and left eye at “near” or “far” distance (Wilcoxon paired samples test, both *p*’s > 0.9). The right dominant eye CMCF data was selected for further statistical analyses. When a right eye CMCF value was missing (8% of cases), it was substituted by a corresponding left eye value to avoid missing values in the analyses.

The individual loading of accommodation at “near” and “far” was calculated by division of the obtained value of the measured accommodative response with the predicted maximum level of accommodation calculated as 25–0.4 × age (Duane 1912).

### Electromyography and electrocardiography

The electrical activity of the upper trapezius muscle was continuously measured bilaterally with bipolar surface electromyography (EMG). Two disposable Ag electrodes (Neuroline 720, Ambu A/S, Ballerup, Denmark) gelled with a 0.5% saline-based electrode paste (GEL101, BIOPAC Systems, Inc., Santa Barbara, CA, USA) were placed on the descending portion of the upper trapezius muscles (center-to-center distance of 20 mm). These electrodes were centered 20-mm lateral to the midpoint of the line between acromion and processus spinosus of vertebra C7, and a grounding electrode was positioned on the processus spinosus of vertebra C7. Before applying the electrodes, the skin was roughened with abrasive paper and cleaned with alcohol. Disposable pre-gelled, general-purpose snap electrodes (EL503, Biopac Systems, Inc., Santa Barbara, CA, USA) for electrocardiography (ECG) were placed on the body bilaterally at the level of the sixth rib.

The EMG and ECG signals were recorded during rest periods, reference contractions, and the tracking task. The EMG and ECG signals were amplified, band-pass filtered (EMG 10–500 Hz, ECG 0.05–35 Hz), and sampled at 2000 Hz (EMG100C, BIOPAC Systems, Inc., Santa Barbara, CA, USA). The ECG signal was used to account for disturbances from heart signals on the raw EMG signal (Zetterberg et al. 2013), in a way similar to the method proposed by Widrow et al. (1975).

**Fig. 1:**
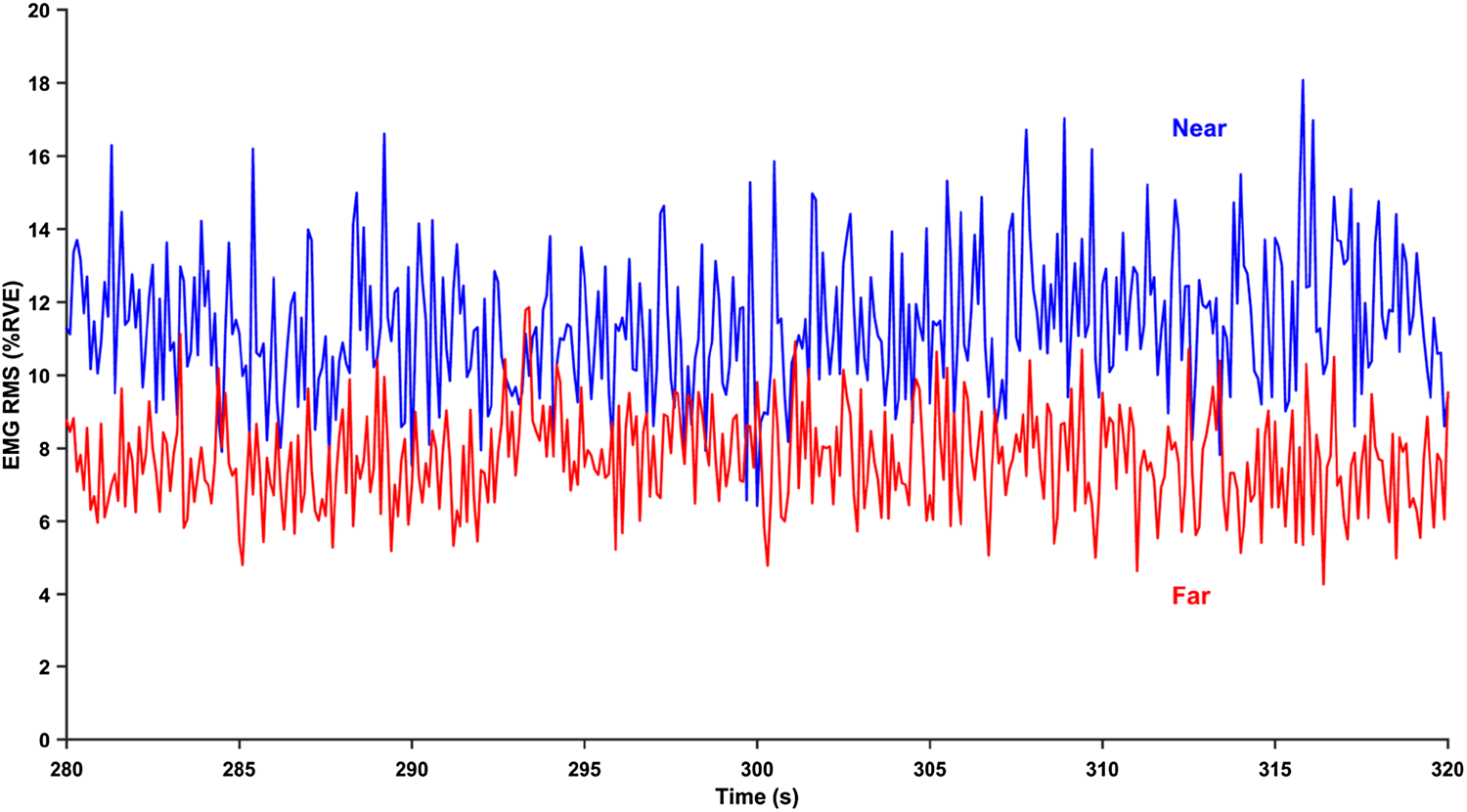
An example of an RMS-converted EMG-recording. The example shows the normalized EMG RMS of the central 40 s from the left trapezius muscle during “far” (upper curve), and “near” (lower curve) screen distances

The EMG recordings were root-mean-square (RMS) converted in 0.1-s periods and adjusted for noise in a power sense. The noise level was estimated as the lowest 400-ms RMS value from the rest period and tracking task period (Thorn et al. 2007; Zetterberg et al. 2013). The RMS values were first computed in microvolts, then normalized to the indivual’s submaximal reference contraction, and expressed in %RVE (reference voluntary electrical activity). During the reference contractions, the subject was asked to hold the arms in 90° abduction with straight elbows and relaxed wrist joints for 15 s (Mathiassen et al. 1995). Figure 1 shows an example of a normalized EMG recording.

The 10th percentile of the normalized RMS values was used to quantify the sustained muscular activity level (Thorn et al. 2007; Zetterberg et al. 2013) of the 1st through the 10th minute and of the full 10 min of the tracking task.

### Data analysis and statistics

Data processing and computation of the tracking error, accommodative response, and EMG parameters were performed with Matlab v.9 (The MathWorks, Inc., USA). Statistical analyses were performed with SPSS v.22 (IBM SPSS Statistics, IBM Corporation, USA). The primary variables for the analyses presented in the “Results” section were accommodation response, ciliary-muscle contraction force, and normalized trapezius EMG. All the variables were tested for normality with the Shapiro–Wilk test of normality. The significance level in all the statistical analyses was set at *p* < 0.05. Depending on the normality of the tested variables, either parametric or non-parametric statistics were used for analysis of each variable (see details in the “Results” section for each specific analysis). When repeated measures ANOVA identified a significant effect among minutes of the task, a post-hoc test of the main effect with Bonferroni correction was applied for pairwise comparisons. To visualize the effect of ciliary-muscle contraction force level on the trapezius EMG level, the generalized estimating equations (GEE) statistical method was applied. The choice of this statistical method was motivated by the fact that GEE relaxes key assumptions of traditional regression models such as normally distributed responses (Ballinger 2004; Fitzmaurice et al. 2011). This analysis uses a correlation-based statistical model to produce mean predicted values of a dependent variable (trapezius EMG activity level) based on the values of a predictor variable (ciliary-muscle contraction force level).

## Results

Tracking error was normally distributed (Shapiro–Wilk test of normality *p* > 0.05 for each screen distance and each minute of the task). Tracking error was significantly larger for “far” distance (1.55 ± 0.38 mm, mean ± SD) than for “near” distance (1.34 ± 0.33 mm) (repeated measures ANOVA on data averaged across minutes, Greenhouse–Geisser *p* < 0.01). Tracking error was not significantly different among the 10 min of the task for “near” screen distance (repeated measures ANOVA, Greenhouse–Geisser *p* = 0.125). For “far” distance, however, there was a trend for increased tracking error over the time of the test. Repeated measures ANOVA showed an overall significance of the trend (Greenhouse–Geisser *p* < 0.01). In pairwise comparisons with Bonferroni correction, the only significant differences in tracking error for “far” distance were between minutes 2 and 7, and minutes 2 and 9 (*p* < 0.05).

**Fig. 2:**
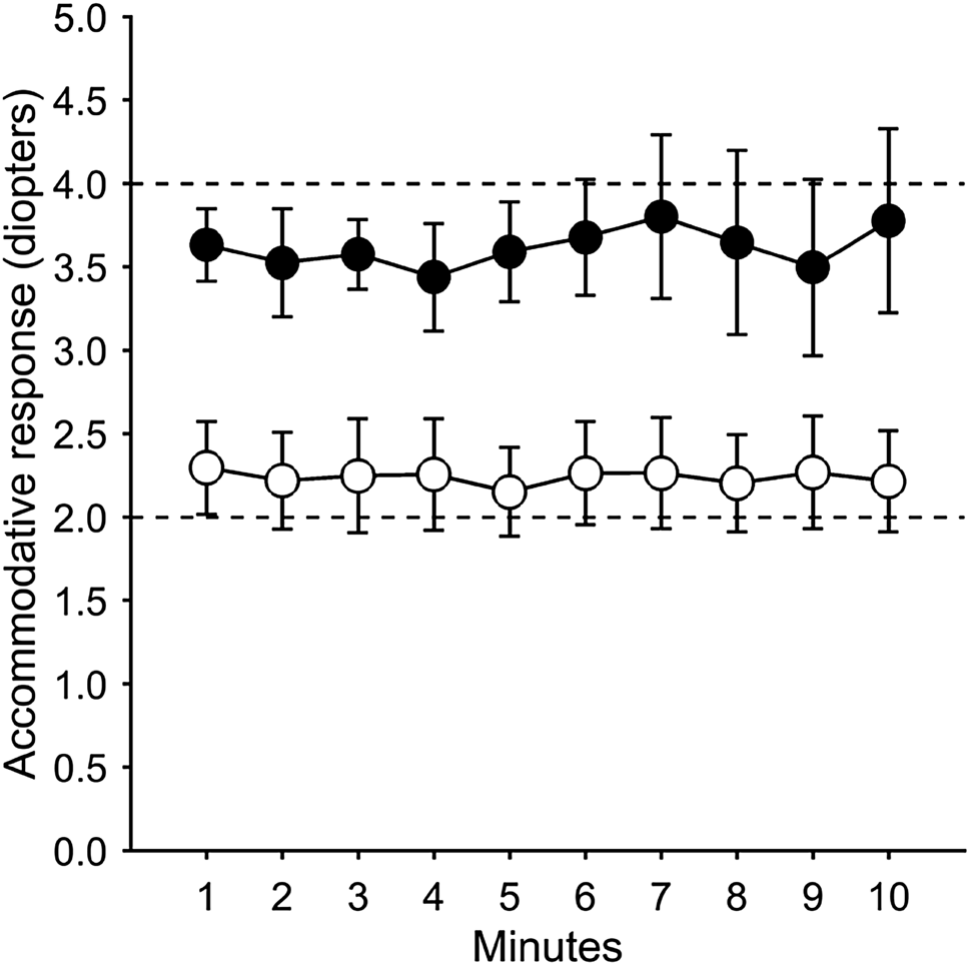
Accommodative response during the time of the test. Dashed lines show reference levels for “far” (2 diopters) and “near” (4 diopters) test conditions. Filled and open circles show accommodative response during “near” and “far” test conditions, respectively. Circles and error bars show mean ± SD for each minute

The accommodative eye-lens response computed across participants for minutes 1 through 10 of the tracking tasks was stable during the time of tracking and was on average 3.6 ± 0.3 (mean ± SD) diopters for “near” and 2.2 ± 0.3 diopters for “far” screen distance (Fig. 2). The accommodation measures exhibited the expected properties, a small lag at “near” and a small lead at “far” (Bernal-Molina et al. 2014; Seidemann and Schaeffel 2003). At “near”, participants loaded their accommodative system with an average of 26% out of age predicted maximum level (min–max 20–46%). At “far”, participants loaded their accommodative system with an average of 16% out of maximum (min–max 12–24%). The accommodative response to the “near” target exhibited an averaged lag of − 0.40 D (min–max − 0.80–0.01; SD 0.27). The accommodative responses to the “far” target showed an averaged lead of 0.27 D (min–max − 0.17–0.95; SD 0.30). Between-participants variability in accommodative response was rather small throughout the task, as evidenced by the standard deviation bars in Fig. 2. The average values of accommodative response for the whole task and for each minute were normally distributed (Shapiro–Wilk test of normality all *p*’s > 0.05). Repeated measures ANOVA identified no significant difference in accommodative response among the 10 min of the task for “near” or “far” screen distance (Greenhouse–Geisser both *p*’s > 0.3).

**Fig. 3:**
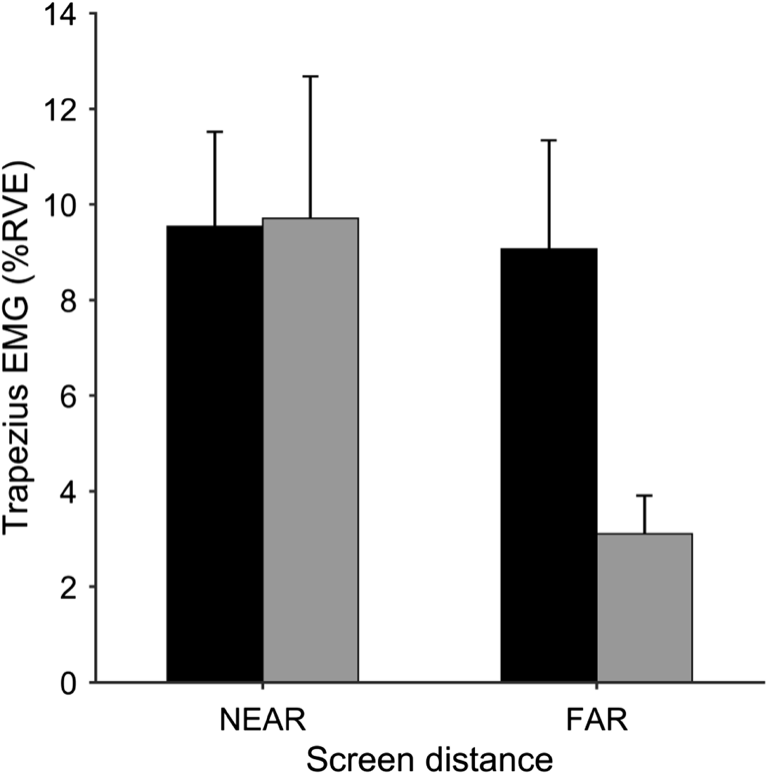
Trapezius EMG for different screen distances and body sides. Black and gray bars show the right and left side, respectively. The bars and error bars show mean ± SEM

The averaged across minutes trapezius EMG values were: left side and “near” screen distance 9.7 ± 9.4% RVE (mean ± SD), left side and “far” distance 3.1 ± 2.5, right side and “near” distance 9.5 ± 6.5, right side and “far” distance 9 ± 7.5 (Fig. 3). The EMG values were not normally distributed (Shapiro–Wilk test of normality *p*’s < 0.05). Wilcoxon paired-samples test on trapezius EMG values for each combination of side and screen distance showed a significant difference in trapezius EMG between left and right side for “far” distance (*p* < 0.01) and between “near” and “far” distance for left trapezius (*p* < 0.05). The trapezius muscle EMG activity on the tracking side was not significantly different from the contralateral passive side for “near” distance (*p* > 0.3). There was no significant difference for the right trapezius EMG for “near” and “far” screen distances (*p* > 0.3).

The Spearman rank correlation analysis between computed ciliary-muscle contraction force (CMCF) and trapezius muscle EMG activity averaged across minutes of the tracking task revealed a significant correlation for the right side at “near” screen distance (*r* = 0.73, *p* < 0.05). The relationship was valid also for nearly every minute of the tracking task for the right side at “near” screen distance: for minutes 1–4 it reached statistical significance (all *r*’s ≥ 0.62, all *p*’s < 0.05) and for minutes 5–8 and minute 10, the relationships bordered on significance (all *r*’s ≥ 0.59, all *p*’s < 0.07). For minute 9, correlation was weaker and did not reach the level of significance (*r* = 0.47, *p* = 0.14). See Table 1.

**Table 1:** Spearman rank correlations between ciliary-muscle contraction force (CMCF) and trapezius muscle EMG activity averaged across minutes of the tracking task for “near” and “far” viewing distance

Viewing distance	Trapezius muscle	Minutes of tracking task										
		1	2	3	4	5	6	7	8	9	10	Grand mean
“Near”	Right side	0.74*	0.64*	0.73*	0.62*	0.59	0.60	0.61	0.60	0.47	0.63	0.73*
	Left side	0.10	0.37	0.25	0.28	0.23	0.28	0.35	0.47	0.43	0.19	0.39
“Far”	Right side	0.64*	0.51	0.64*	0.44	0.04	0.08	0.07	0.19	0.51	0.52	0.10
	Left side	0.37	0.44	0.26	− 0.19	− 0.07	− 0.14	− 0.21	− 0.26	− 0.43	− 0.25	− 0.26

*p < 0.05

The right side and “far” screen distance correlation between CMCF and trapezius EMG averaged across minutes was not significant (*r* = 0.1, *p* = 0.7). For minutes 1 and 3, however, it reached statistical significance (*r*’s = 0.64, *p*’s < 0.05).

For the left side, either for “near” or “far” screen distance, there were no significant correlation between CMCF and left trapezius EMG (all *p*’s > 0.05).

**Fig. 4:**
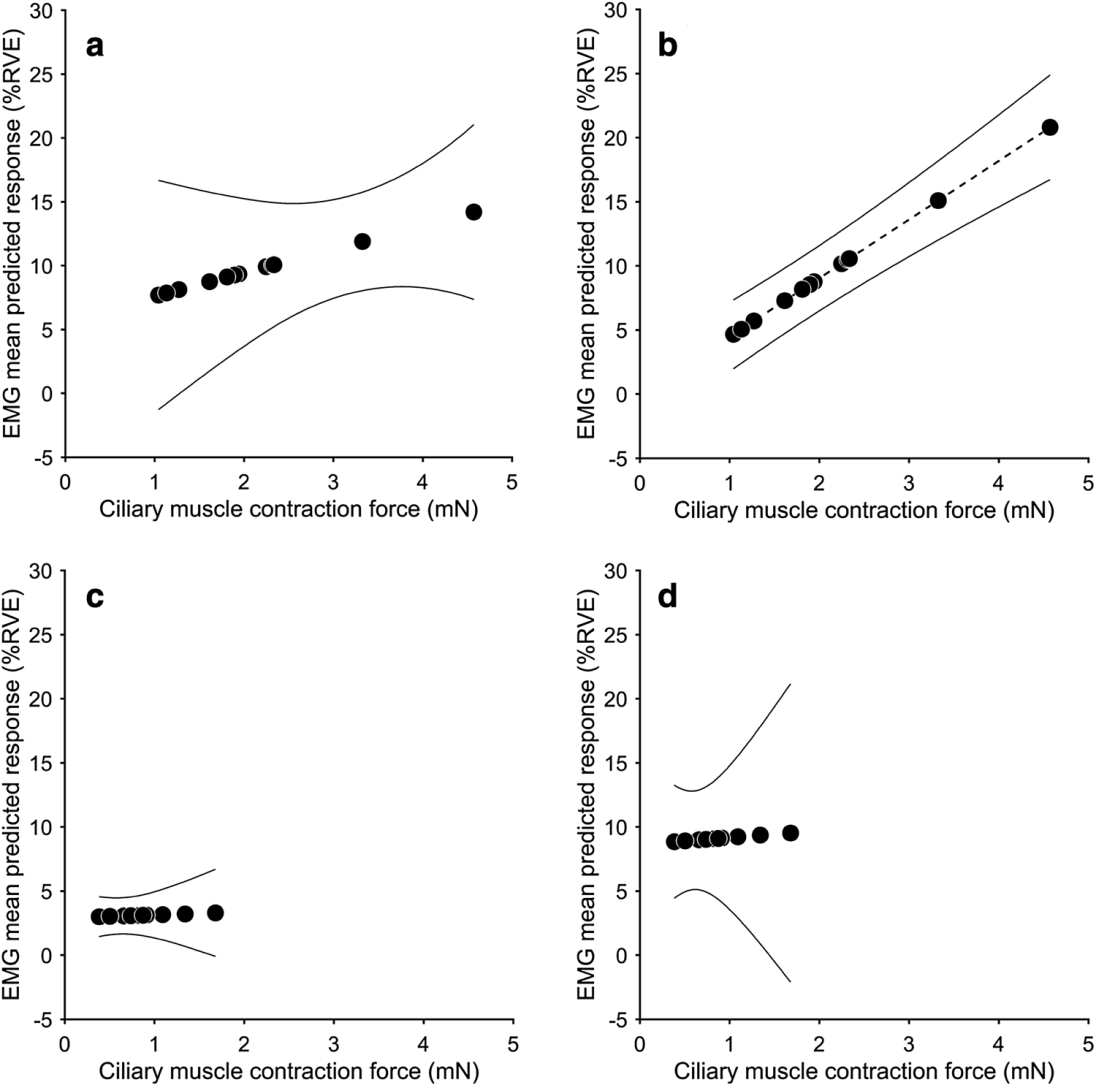
Mean predicted response of trapezius EMG from GEE analysis. **a** Left side, “near” screen distance. **b** Right side, “near” screen distance. **c** Left side, “far” screen distance. **d** Right side, “far” screen distance. The GEE model shows a significant effect only in **b** (indicated by dashed regression line). Solid lines show upper and lower 95% confidence intervals

The generalized estimating equations (GEE) model was significant only for the right trapezius and “near” screen distance combination (*p* < 0.001). For the other three combinations of screen distance and EMG-side, the model was not significant, with *p* values 0.9 (right side and “far” distance), 0.36 (left side and “near” distance), and 0.88 (left side and “far” distance). The mean predicted values for trapezius EMG levels are shown in panels of Fig. 4.

## Discussion

In this study, the relationship between ciliary-muscle contraction force and trapezius muscle activity was studied during a free gaze viewing setting. Two different viewing conditions both necessitated continuous accommodation of the eye-lens during concurrent performance of a dynamic eye–hand work task. The main finding was a strong and significant correlation between the level of ciliary-muscle contraction force and the level of trapezius muscle activity on the active tracking side, at the closer viewing distance. That is, participants who had higher levels of ciliary-muscle contraction force, at the nearest viewing distance, also demonstrated higher levels of trapezius muscle activity on the active side during performance of the eye–hand work.

### A functional relationship between ciliary-muscle contraction force and trapezius muscle activity

The correlation between ciliary-muscle contraction force and trapezius muscle activity on the active side at the “near” distance, as discussed in previous studies, is likely attributed to a generic motor command of central origin (Richter et al. 2010). This motor command to the two effectors (eye muscles and arm/hand muscles) was parallel, simultaneous, and complementary, i.e., it produced different mechanical effects on different anatomical structures, effects that converged in obtaining the composite result of bringing the target image to focus and optimizing tracking motor performance. The individual level of trapezius muscle activation on the active side at the “near” distance, which was associated with the magnitude of the eye-lens accommodation response, may be attributed to the reflex optic paths at the origin not only of the ocular response but also of the extraocular, neck, and scapular muscles as well.

For the right active side at “far” a weak tendency to control synergy emerged. However, the overall correlation was non-significant. The lack of a significant correlation between ciliary-muscle contraction force and trapezius muscle activity on the active side at the “far” screen distance is likely related, directly, to the lower level of accommodation load at this distance. At “far”, the level of accommodation load was substantially lower if compared to “near”, i.e., respectively, 16% compared to 26%. The reason for this rather low level of accommodative load is related to the young age of the participants and to the design of the current study. An even stronger relationship is expected between eye–neck effectors in occupational settings at closer work distance and with oculomotor load levels closer to maximum and during completely free head movements. However, such exposures were not possible to achieve in this study due to limitations of the eye refraction measurement methodology.

Hence, a prerequisite for the correlation between ciliary-muscle contraction force and trapezius muscle activity on the side performing the motor task seems to be accommodative load or accommodative effort above a critical threshold level together with a minimal amount of muscle “involvement” (muscle effort and muscle effector system complexity). At the “near” screen distance, the visual load was already rather low and at “far” distance the visual load was likely below this critical threshold level. In both viewing conditions the muscle “involvement” was rather low, i.e., operating a computer mouse placed on the table resulted in rather low levels of trapezius muscle activity on the active side. The control of the effector system in this study had a smaller number of degrees of freedom than that for the tracking of a visual target with a pen and without hand support, as in the previous study by Domkin et al. (2016). It can be thought that when accommodative load, muscle load, and control complexity reach a certain critical level, the eye–head–neck–arm system transforms into one functional unit to achieve sufficient gaze stabilization for appropriate motor performance.

In terms of magnitude, the average level of the right trapezius EMG (active side) was similar in both viewing conditions (“near” and “far”) and reflected the required low level of muscle activation to perform the computer mouse work, which was identical in both conditions. Despite similar trapezius EMG magnitudes and similar complexity of the control of the motor system on the active side for “near” and “far” conditions, the nearer viewing distance and thus the corresponding higher visual load triggered the sensorimotor synergy between trapezius muscle activity and ciliary muscle contraction force, as discussed above. The magnitude of the left trapezius EMG (passive side) was lower than that of the right trapezius EMG at “far” distance. This is expected since the left side was not involved in the motor task. The left trapezius EMG was, however, higher for “near” distance than for “far” distance and its level for “near” distance was similar to the level of the right trapezius EMG. The increase in accommodation at “near” may have resulted in an increase in the left trapezius EMG by general mechanisms acting on both sides, even if the left side did not perform the task. However, for the left side, it did not result in the emergence of a correlation between trapezius muscle activity and ciliary muscle contraction force likely due to the lack of necessity of control of the motor system of the side that did not participate in the motor task.

The results of this study do not point to a personal “eye–neck trait” (i.e., a genetic or learned pattern of always coupling a higher level of trapezius muscle activity with a higher level of eye-lens accommodation or vice versa) as the reason for the current correlation between ciliary-muscle contraction force and trapezius muscle activity. A trait or habitual pattern of oculomotor function ought to manifest itself equally strong at all viewing distances regardless of the level of oculomotor load. Thus, if the trait existed, the trapezius EMG and ciliary-muscle contraction force would be expected to correlate at both (“near” and “far”) screen distances. However, such a correlation was only demonstrated at the nearest distance.

### Methodological considerations and limitations

The larger tracking error for “far” condition could be attributed, at least partly, to the size of the mouse cursor that was constant in both conditions. The cursor consequently appeared smaller in the “far” condition and thus it may have been more difficult for participants to keep its tip in the middle of the target in the same way they did in the “near” condition. This circumstance may also be the reason for the increase in tracking errors over time which occurred only during tracking at “far”.

The design of the current study was also influenced by methodological limitations of the autorefractor which necessitates a pupil diameter of at least 3 mm or larger. A higher level of ciliary-muscle contraction force, than what was used in the current study, would have been desirable. However, if the pupil diameter is smaller than 3 mm [which can often be the case for higher levels of accommodation load (Ciuffreda 1998)], then the autorefractor signal is not registered. This methodology also requires alignment of the photorefractor axis and axes of the eyes, as well as a constant eye–screen distance, which inevitably demands restricted head movement. In the scientific literature, methods have been suggested that could overcome this limitation, e.g., impedance cyclography (Swegmark and Olsson 1968).

## Conclusion

The current study confirmed earlier findings and showed that sustained levels of ciliary-muscle contraction force, during free gaze conditions and concurrent hand/arm movements, correlate with trapezius muscle activation levels across individuals. By disproving the notion that a personal trait drives the eye–neck correlation, this study extends the knowledge of and provides insight into the underlying mechanisms that link the eyes with the neck. The current results suggest that a ciliary-muscle contraction force above a critical threshold level is required to observe this correlation. By use of an occupationally relevant approach (i.e., computer mouse work) the results from this study can be generalized to a wider range of occupational settings than that reported previously.

## Abbreviations

CMCF: Ciliary-muscle contraction force
Diopter (D): The refractive power of a lens or an optical system. It is the reciprocal of distance in meters so that 1 diopter corresponds to 1 m, 2 diopters corresponds to 0.50 m, etc.
ECG: Electrocardiography
EMG: Electromyography
RMS: Root mean square
RVE: Reference voluntary electrical activity
